# RAGE Inhibition Reduces Surgery-Induced Cerebral Edema After Glioma Resection

**DOI:** 10.1227/neu.0000000000003987

**Published:** 2026-03-19

**Authors:** Mojtaba Dayyani, Aleksandr Filippov, Ian Zhang, Joseph Georges, Zaan Saeed, Jillyn Turunen, Nick Sobhanian, Huiling Yuan, Massimo D’Apuzzo, Yue Hao, Michael E. Berens, Leying Zhang, Jana Portnow, Behnam Badie

**Affiliations:** 1 Division of Neurosurgery, City of Hope Beckman Research Institute, Duarte, California, USA; 2 Department of Pathology, City of Hope Beckman Research Institute, Duarte, California, USA; 3 Clinical Genomics and Therapeutics Division, Translational Genomics Research Institute, Phoenix, Arizona, USA; 4 Department of Medical Oncology, City of Hope Beckman Research Institute, Duarte, California, USA

**Keywords:** Brain edema, Corticosteroids, Glioblastoma, Surgical brain injury, Vasogenic edema

## Abstract

**BACKGROUND AND OBJECTIVES:**

Cerebral edema (CE) is a common contributor to neurological decline after brain tumor resection. While corticosteroids are effective in managing CE perioperatively, their use is associated with significant side effects and potential interference with immunotherapeutic efficacy in patients with malignant brain tumors. This study aimed to evaluate the anti-inflammatory effects of 2 inhibitors of the receptor for advanced glycation end products (RAGE)—TTP488 and FPS-ZM1—on CE development after glioma resection in murine models.

**METHODS:**

Mice bearing orthotopic CT-2A gliomas were randomized into 4 treatment groups before undergoing fluorescence-guided microsurgical tumor resection. The groups received perioperative administration (from day −4 to day +7) of TTP488, FPS-ZM1, dexamethasone, or vehicle. Postoperative CE was assessed using serial brain MRI over a 7-day period and quantified using manual segmentation. Neurological function, wound healing, and response to anti–PD-1 immunotherapy were also evaluated. Bulk RNA sequencing was performed to analyze differential gene expression associated with RAGE inhibition.

**RESULTS:**

Across all groups, CE peaked on postoperative day 2 and subsided by day 7. On postoperative day 1, both TTP488 and FPS-ZM1 significantly reduced CE compared with vehicle (*P* = .03 for TTP488; *P* = .03 for FPS-ZM1). Notably, unlike dexamethasone, neither RAGE inhibitor impaired the efficacy of anti–PD-1 immunotherapy. FPS-ZM1 treatment was also associated with improved neurological recovery, enhanced wound healing, and potentiated anti–PD-1 therapy at higher doses.

**CONCLUSION:**

RAGE inhibitors effectively reduced postoperative CE to a degree comparable with dexamethasone, without compromising the efficacy of immunotherapy or wound healing. These findings suggest that RAGE inhibition may offer a promising steroid-sparing strategy for perioperative management of CE in patients with brain tumor undergoing immunotherapy.

Since its introduction decades ago,^1^ dexamethasone has been routinely administered perioperatively to manage cerebral edema (CE) resulting from surgical brain injury (SBI), which typically persists for 2 to 3 weeks after craniotomy.^2–4^ Postoperative CE significantly contributes to patient morbidity and neurological deterioration during the perioperative period.^5,6^ Although dexamethasone is an effective and low-cost anti-inflammatory agent, it is profoundly immunosuppressive and impairs the body’s natural antitumor immune responses.^7^ Moreover, its use is associated with a range of adverse effects, including peptic ulcers, hyperglycemia, impaired wound healing, increased infection risk, and psychosis, that may negatively affect surgical outcomes.^8^ In patients with malignant brain tumors, corticosteroids have been shown to diminish the efficacy of immunotherapies.^9^ A secondary analysis of the CheckMate 143 Phase 3 trial of a PD-1 inhibitor in recurrent glioblastoma revealed that concurrent dexamethasone use was associated with worse survival outcomes.^9^ With the emergence of novel immunotherapeutic strategies such as oncolytic viruses and chimeric antigen receptor T cells,^10–12^ there is an urgent need for alternative therapies that can control postoperative CE without compromising immunotherapy efficacy.

CE after SBI arises from both vasogenic and cytotoxic mechanisms. Vasogenic edema results from the disruption of endothelial tight junctions because of oxidative stress and inflammation.^13^ Activated glial cells increase blood-brain barrier (BBB) permeability by releasing vascular endothelial growth factors and matrix metalloproteinases, causing protein/fluid extravasation and extracellular water accumulation.^13^ SBI triggers the release of cytokines and chemokines that activate astrocytes and microglia promoting the recruitment of myeloid-derived cells.^2,14–16^ In addition, damage-associated molecular patterns activate Receptor for Advanced Glycation End products (RAGE) on leukocytes,^14^ with ligands such as S100 calcium-binding protein B, S100 calcium-binding protein A9 (S100A9), and high mobility group box 1 initiating central nervous system proinflammatory cascades after brain ischemia and injury.^17–20^

In previous work, we demonstrated that activation of RAGE by S100A9, a ligand released by infiltrating neutrophils and monocytes, induces neuroinflammation after brain injury.^21^ Inhibition of the RAGE–S100A9 axis was as effective as dexamethasone in reducing CE in a murine SBI model. Based on these findings, we hypothesized that RAGE inhibitors could also alleviate CE associated with tumor resection surgery.

To test this hypothesis, we evaluated the anti-inflammatory effects of 2 RAGE inhibitors, TTP488 (azeliragon) and FPS-ZM1, in a murine glioma resection model. TTP488 is an orally bioavailable small molecule RAGE inhibitor that has shown beneficial effects in preclinical models of Alzheimer’s disease.^22,23^ Although it failed to improve cognitive outcomes in clinical trials for mild Alzheimer’s disease, it was well-tolerated and exhibited minimal toxicity.^23,24^ FPS-ZM1 is another brain-penetrant RAGE inhibitor that reduces proinflammatory cytokine expression in models of stroke and SBI.^21,25,26^

In this article, we demonstrate the efficacy of RAGE inhibitors as steroid-sparing alternatives for perioperative CE management after glioma resection and their compatibility with immunotherapy and wound healing.

## METHODS

The effect of TTP488 and FPS-ZM1 on CE was investigated in in vivo mouse models of SBI and tumor resection survival surgery. Furthermore, additional experiments assessed the effects of RAGE inhibitors on anti–PD-1 immunotherapy and acute postoperative wound healing. All animal care and experiments complied with the Guide for the Care and Use of Laboratory Animals^27^ and were approved by our Institutional Animal Care and Use Committee Protocol #09015.

### Reagents and Cell Lines

Murine CT-2A glioma and Raschke-Abelson-Wetzell monocyte lines were purchased from American Type Culture Collection. Both cells were cultured in supplemented Dulbecco’s modified Eagle’s medium as described before.^21^ Details of all reagents are given in Supplemental Digital Content 1 (**Reagents and Cell Lines**, http://links.lww.com/NEU/F287).

### NF-κB and IFN Activation Assays

Comprehensive descriptions of THP-1 Quanti-Blue and THP-1 Renilla luciferase assays are provided in Supplemental Digital Content 1 (**NF-κB and IFN Activation Assays**, http://links.lww.com/NEU/F287).

### SBI Model

**FIGURE 1.**
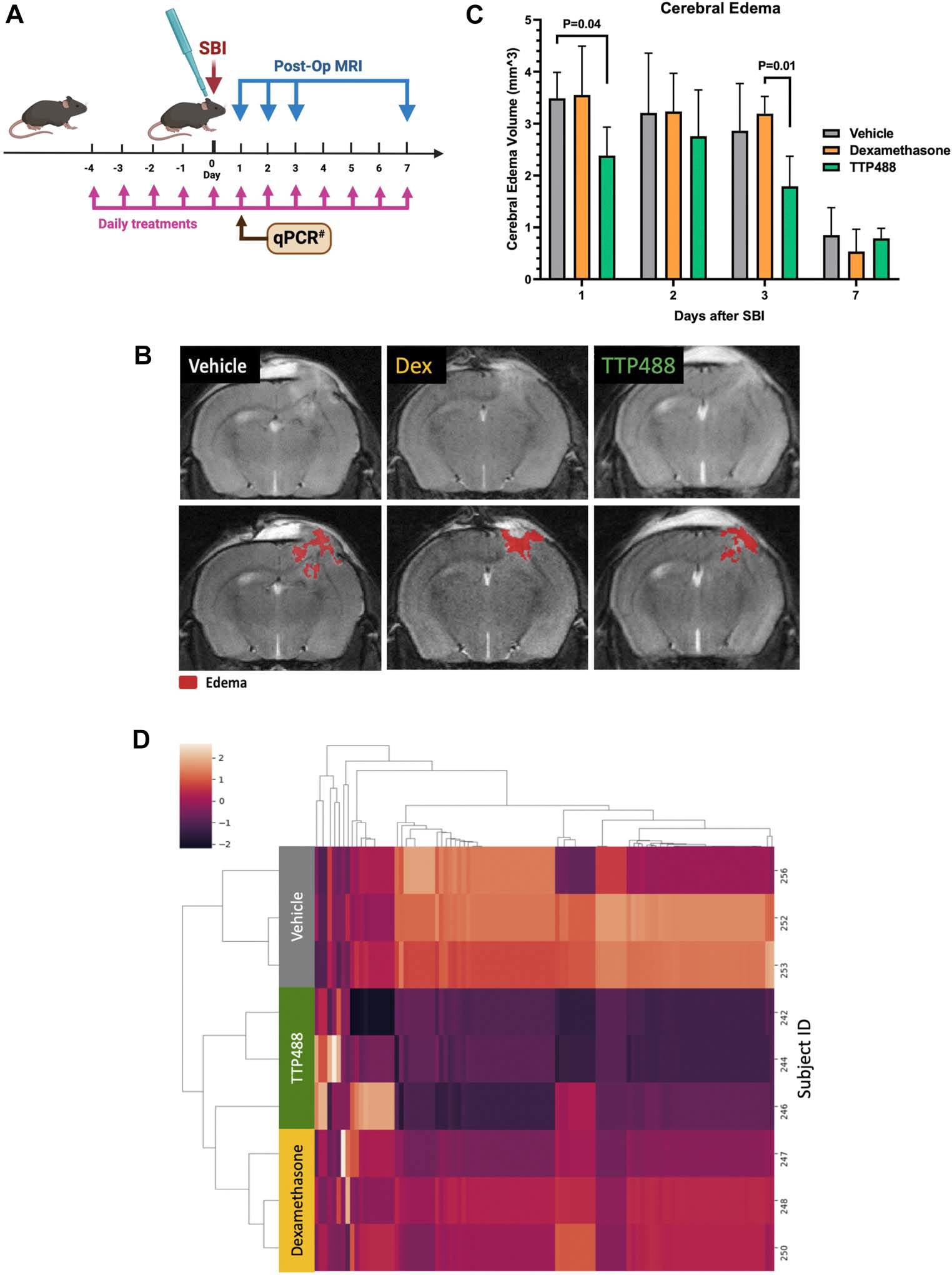

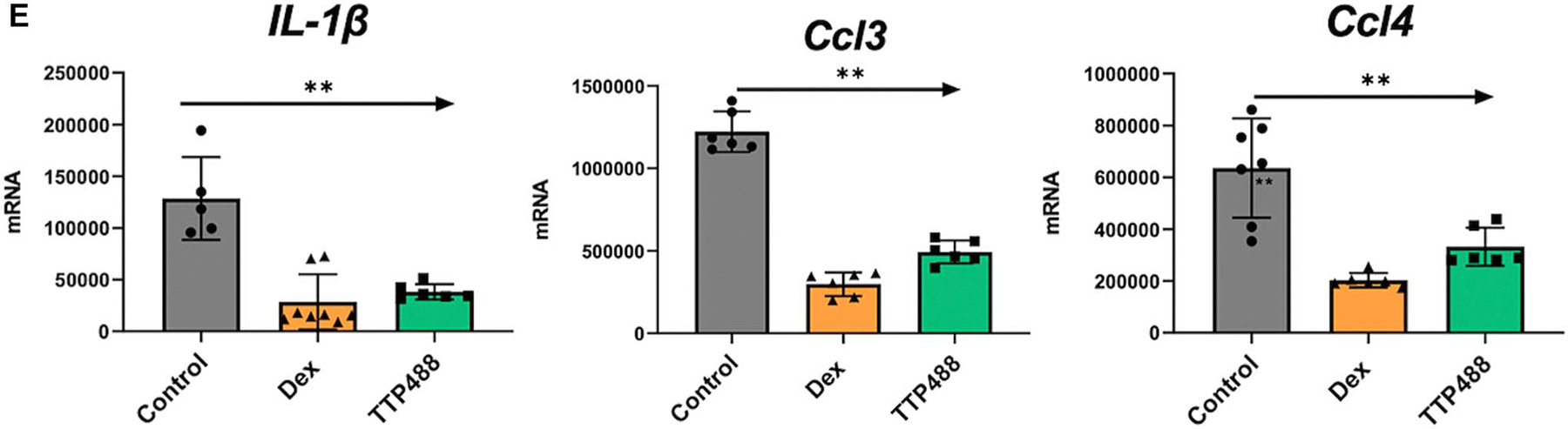
TTP488 abrogates cerebral edema after SBI. **A**, Experimental schema illustrating the therapeutic and diagnostic intervention timelines. Mice (n = 4/treatment group) began receiving daily vehicle (100 μL, IP), dexamethasone (100 μg, IP), or TTP488 (100 μg, IP) 4 days before SBI and until termination of the study. **B**, Representative images of manual segmentation and contouring of the CE on axial MRIs (T2-weighted image, POD 3) of the 3 treatment groups. **C**, Quantification of the CE based on volumetric analysis of MRI voxel counts showed that TTP488 was more effective than vehicle on POD 1 and more effective than dexamethasone on POD 3 (2-way analysis of variance, F = 40.44, P < .0001; post hoc Dunnett’s multiple comparison test: TTP488 vs vehicle P = .04, and TTP488 vs dexamethasone P = .01) and controlled CE as good as dexamethasone on all postoperative days. **D**, Radiomic feature clustering of CE changes after SBI on POD 1 revealed a closer relationship between dexamethasone and TTP488 compared with the vehicle, indicating the similarity of the radiological phenotypes for these 2 agents. **E**, qPCR revealed that the expression of proinflammatory cytokines/chemokines was significantly dampened by TTP488 after SBI. n = 6 mice/group, mean ± SD (**P < .01; analysis of variance). CE, cerebral edema; Dex, dexamethasone; IP, intraperitoneal; POD, postoperative day; Post-Op, postoperative; qPCR, real-time quantitative polymerase chain reaction; SBI, surgical brain injury. Created in BioRender. Dayyani, M. (2026) https://BioRender.com/d8cm24o.

SBI was induced as described.^21^ Briefly, mice were treated perioperatively with TTP488 (100 μg, intraperitoneal [IP]), dexamethasone (100 μg, IP), or vehicle (100 μL, IP), followed by craniectomy and brain tissue excision, with CE quantified by serial MRI on postoperative days (PODs) 1, 2, 3, and 7 (Figure 1A and 1B and Supplemental Digital Content 1, **Surgical Brain Injury Model**, http://links.lww.com/NEU/F287).

### Brain Tumor Resection Survival Surgery Model

Mice (C57BL/6J) harboring 10-day-old CT-2A gliomas were imaged using MRI to assess tumor size and location. Mice with superficial tumors that were amenable to resection were then stratified into uniform treatment groups. Because of the complexity of the procedure and the heterogeneity of tumor location and size, this experiment was conducted in 2 phases. The first experiment compared the activity of TTP488 with that of control and dexamethasone-treated mice (n = 4/group). After validating the imaging parameters, the second experiment compared the activity of FPS-ZM1 (2 mg, IP) and TTP488 (100 μg, IP) with that of vehicle (100 μL, IP) and dexamethasone-treated (100 μg, IP) mice (n = 6/group). In each experiment, treatments began 4 days before fluorescence-guided glioma resection survival surgery with a novel U-arm neurosurgical platform and were continued for 7 days postprocedure. CE was quantified by serial brain MRIs on PODs 1, 2, 3, and 7. Data from the 2 experiments *were* combined for analysis. The protocol for fluorescence-guided glioma resection survival surgery is provided in Supplemental Digital Content 1 (Fluorescence-Guided Glioma Resection Survival Surgery, http://links.lww.com/NEU/F287).

### Immunotherapy and Dexamethasone Administration Protocol

Weekly injections of anti–PD-1 (100 μg, IP, #BP073 BioXCell) or isotype IgG control (100 μL, IP, #BE0089, BioXCell) were administered 7 days after tumor implantation. Daily TTP488 (100 mg/kg, IP) and FPS-ZM1 (50 or 100 mg/kg, IP) therapies were initiated 4 days after tumor implantation and continued for up to 21 days. TTP488 and FPS-ZM1 doses were selected based on previous publications in mouse models.^21,28^ In SBI, tumor resection, and wound healing experiments, 100 μg of dexamethasone was administered, which is equivalent to 4 mg/kg and is considered a high dose in humans.^29^

### MRI Protocol, Image Preprocessing, and Segmentation

A 7-T small animal MRI system (MR Solution, Inc.) was used to verify tumor engraftment one day before tumor resection surgery and to calculate tumor volumes and the extent of CE on PODs 1, 2, 3, and 7 using T1-weighted image, T2-weighted image, fluid attenuated inversion recovery, contrast-enhanced T1-weighted image, and delayed contrast-enhanced T1-weighted image (10 minute-delayed) imaging. The imaging specifications are given in Supplemental Digital Content 1 (Supplemental Table 1, http://links.lww.com/NEU/F287). Images were segmented for tumor, resection cavity, ventricles, total brain tissue, CE, and residual tumor using Insight Toolkit - segmentation and N-dimensional application package version 3.8.0.^30^ Volumes/masks were manually segmented and are displayed in Supplemental Digital Content 1 (Supplemental Figure 1, http://links.lww.com/NEU/F287), along with their accompanying heuristics. Radiomic clustering analysis of CE after SBI was performed according to the methodology outlined in Supplemental Digital Content 1 (**Radiomic Cluster Analysis**, http://links.lww.com/NEU/F287, and **Radiomic Feature Extraction Settings**, http://links.lww.com/NEU/F287).

### Postoperative Neurological Function and Recovery Assessment

Simple Neuroassessment of Asymmetric Impairment (SNAP) was used to evaluate the neurological function of the mice on PODs 1, 3, and 7. The SNAP score comprises 8 separate tests that assess vision, proprioception, motor strength, and posture using a 5-level scoring system, ranging from 0 to 5.^31^

### Wound Healing Assessment

In pilot and complementary experiments, linear and round scalp wound models in mice were used to evaluate wound healing through blinded scoring, ImageJ-based quantification, and histological evaluation. Comprehensive protocols of wound healing experimental design and assessment are provided in Supplemental Digital Content 1 (**Comprehensive Wound Healing Experimental Design**, http://links.lww.com/NEU/F287, and **Wound Healing Assessment Protocol**, http://links.lww.com/NEU/F287).

### Real-Time Polymerase Chain Reaction

Real-time quantitative polymerase chain reaction was performed using corresponding primers (Supplemental Digital Content 1, Supplemental Table 2, http://links.lww.com/NEU/F287) and a TaqMan 5700 Sequence Detection System (Applied Biosystems) as described.^32^

### Bulk RNA-seq Data Analysis

Bulk RNA-seq sequencing was performed on THP-1–derived macrophages according to the protocol by Rynikova et al.^33^ In brief, THP-1 monocytes were first differentiated into M0 macrophages (phorbol 12-myristate 13-acetate, 25 ng/mL for 48 hours). The phorbol 12-myristate 13-acetate media were replaced with fresh media for 24 hours before treating the cells with lipopolysaccharide (50 ng/mL) + IFN-γ (5 ng/mL, M1), IL-4 (25 ng/mL, M2), S100A9 (500 ng/mL), and each RAGE inhibitor (TTP488: 5 μM; FPS-ZM1: 50 μM) for another 24 hours. Transcriptomics FASTQ data quality was assessed using FastQC v0.11.8^34^ and preprocessed using fastp.^35^ A detailed protocol for bulk RNA-seq analysis can be found in Supplemental Digital Content 1 (**Bulk RNA-Seq Data Analysis**, http://links.lww.com/NEU/F287).

### Statistical Analysis

Statistical comparisons including one-way and two-way analysis of variance and mixed-effect model analysis were performed as appropriate using GraphPad Prism 9 (GraphPad Software, Inc.). Dunnett’s multiple comparison test was used as post hoc analysis. Two-sided *P* < .05 was considered significant. Survival was determined using Kaplan–Meier graphs and compared using the log-rank test and Cox regression. Interclass correlation coefficient was calculated using SPSS Version 22.0 (SPSS Software, Inc.).

### Data Availability

All data are freely available in Supplemental Digital Content 1 (http://links.lww.com/NEU/F287). No additional data are available.

## RESULTS

### TTP488 Suppresses CE Formation and Inflammation After SBI

Previously, we demonstrated that FPS-ZM1 inhibited neuroinflammation and BBB disruption (as indicated by Evans Blue extravasation) in a murine SBI model.^21^ Before investigating the effect of RAGE inhibitors in glioma models, we validated the utility of MRI for CE quantification by repeating the SBI experiments with TTP488 (Figure 1A and 1B). After unilateral SBI, CE increased by day 2, followed by a rapid decrease to baseline by day 7 (Figure 1C). Although mice treated with TTP488 exhibited lower CE than vehicle and dexamethasone groups, this reduction was only statistically significant on POD 1 and POD 3 when compared with the vehicle (*P* = .04) and dexamethasone (*P* = .01), respectively.

In addition, clustering of the radiomic features revealed a marked decrease in inflammatory parameters in the TTP488-treated group (Figure 1D). To compare the inflammatory responses to SBI, brains from mice treated with TTP488, dexamethasone, or vehicle were collected 24 hours after unilateral SBI and assessed for the expression of proinflammatory cytokines and chemokines by the real-time quantitative polymerase chain reaction. As previously reported for FPS-ZM1,^21^ the expression of proinflammatory cytokine (*IL-1β*) and chemokines (*Ccl3* and *Ccl4*) was significantly inhibited by TTP488 (Figure 1E). These findings confirmed the anti-inflammatory effects of another RAGE inhibitor (TTP488) and validated MRI as a clinically relevant tool to measure CE in the murine SBI model.

### RAGE Inhibitors Reduce CE After Brain Tumor Resection

**FIGURE 2.**
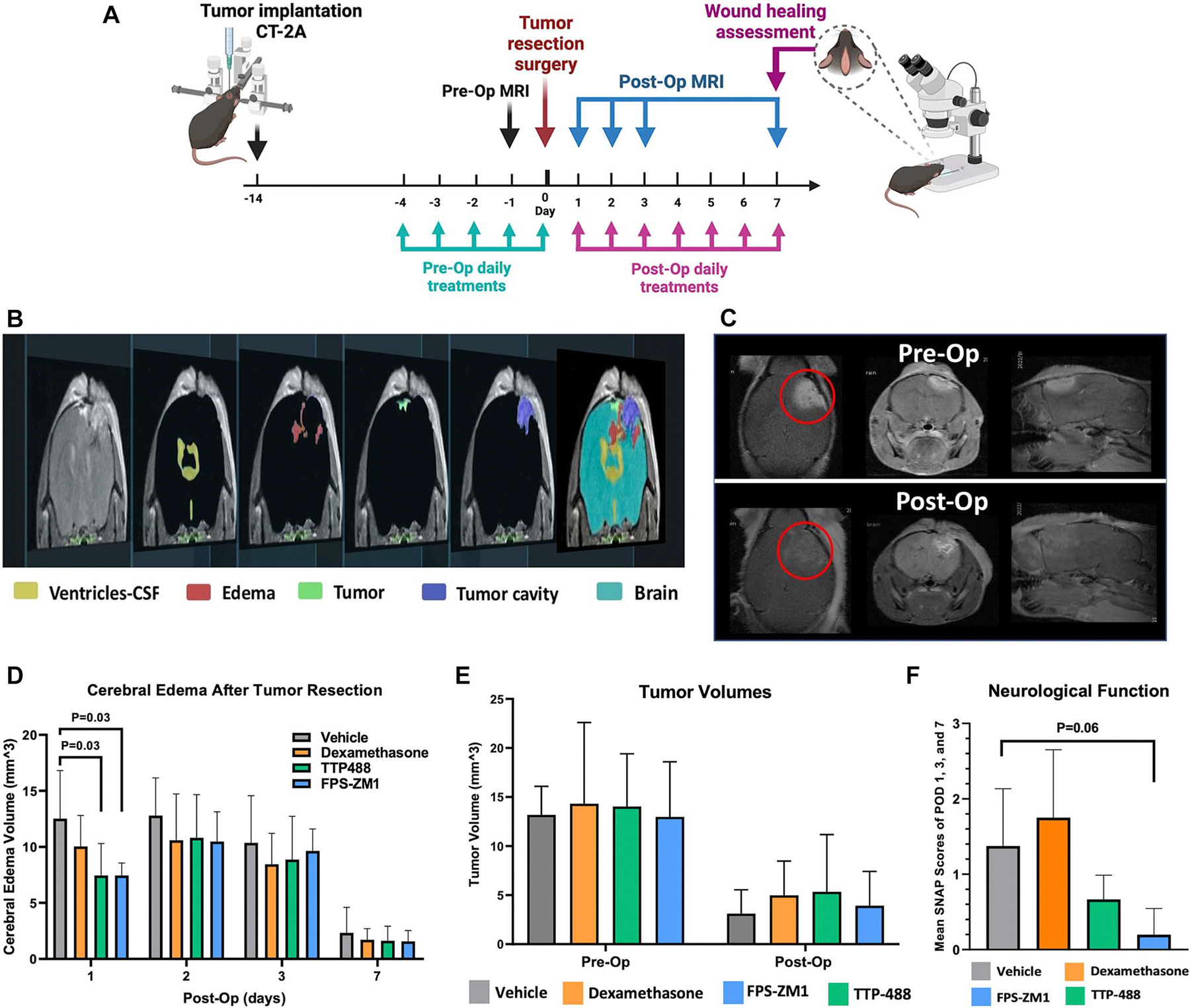
Receptor for Advanced Glycation End products inhibitors reduce cerebral edema after brain tumor resection. **A**, Design of the tumor resection experiment illustrating the timeline of the therapeutic, surgical, and diagnostic interventions. Mice received daily vehicle (100 μL, IP, n = 10), dexamethasone (100 μg, IP, n = 10), TTP488 (100 μg, IP, n = 10), or FPS-ZM1 (2 mg, IP, n = 6). **B**, Representative post-op brain MRIs demonstrating the segmented axial planes of the resection cavity, ventricles, total brain tissue, cerebral edema, and residual tumor using ITK-SNAP software. **C**, Coronal, axial, and sagittal contrast-enhanced T1-weighted MRI images of a representative mouse, before and after a successful tumor resection surgery. **D**, Quantification of the CE volume (mm^3^ ) on segmented MRIs demonstrated that both TTP488 and FPS-ZM1 significantly reduced CE on POD 1 (mixed-effect model analysis, F = 88.81, P < .0001; post hoc Dunnett’s multiple comparison test for vehicle vs TTP488 and vehicle vs FPS-ZM1, P = .03). **E**, The pre- and postoperative volumetric measurements of the tumor size revealed no differences among the treatment groups, indicating sufficient similarity among the study subjects. **F**, Assessment of the postoperative neurological function demonstrated an improvement of SNAP score (lower value indicates better function) by FPS-ZM1 over a 7-day postoperative period (2-way analysis of variance, F = 7.55, P = .002; post hoc Dunnett’s multiple comparison test, P = .06). CE, cerebral edema; CSF, cerebrospinal fluid; IP, intraperitoneal; ITK-SNAP, Insight Toolkit - segmentation and N-dimensional application package; POD, postoperative day; Pre-Op, preoperative; Post-Op, postoperative; SNAP, Simple Neuroassessment of Asymmetric Impairment. Created in BioRender. Dayyani, M. (2026) https://BioRender.com/d8cm24o.

Similar to SBI, the quantification of the CE in manually segmented MRIs (Figure 2A–2C) revealed that brain edema peaked on POD 2 and gradually diminished by day 7 after tumor resection. On POD 1, mice treated with TTP488 and FPS-ZM1 showed a marked reduction of CE compared with the vehicle group (*P* = .03, TTP488 vs vehicle; *P* = .03, FPS-ZM1 vs vehicle) (Figure 2D). To exclude the possible confounding effect of baseline variability in tumor size and the extent of tumor resection on postoperative CE quantification, we compared the preoperative tumor and postoperative residual tumor volumes among the groups and found no significant variations (Figure 2E).

### RAGE Inhibitors Improve Postoperative Neurological Function

The FPS-ZM1 group had the lowest mean SNAP score (indicating better neurological function) compared with the vehicle group through POD 7 (*P* = .06) (Figure 2F). Similarly, the TTP488-treated mice exhibited better neurological function than the vehicle and dexamethasone groups through POD 7; however, the difference between the groups was not statistically significant (Figure 2F). Considering that the SNAP is a measure of neurological recovery rate cumulatively assessed over 7 days, our findings indicate that control and dexamethasone-treated mice that had higher CE recovered more slowly from CE-induced brain injury that occurred during the early phases of SBI compared with both RAGE inhibitors. Supplemental Digital Content 1 (Supplemental Figure 2, http://links.lww.com/NEU/F287) shows representative images of positive and negative Baton test.

### RAGE Inhibitors Do Not Affect Wound Healing

In a pilot study, we first compared the effect of TTP488 with clinically relevant doses of dexamethasone on the healing of a closed linear wound. TTP488-treated mice had significantly lower scores for redness compared with vehicle and dexamethasone-treated mice. Dehiscence scores were highest for the dexamethasone group, and a statistically significant decrease in dehiscence scores was seen for TTP488-treated mice compared with those treated with dexamethasone (*P* = .00001) or vehicle (*P* = .00003) (Supplemental Digital Content 1, Supplemental Figure 3, http://links.lww.com/NEU/F287).

**FIGURE 3.**
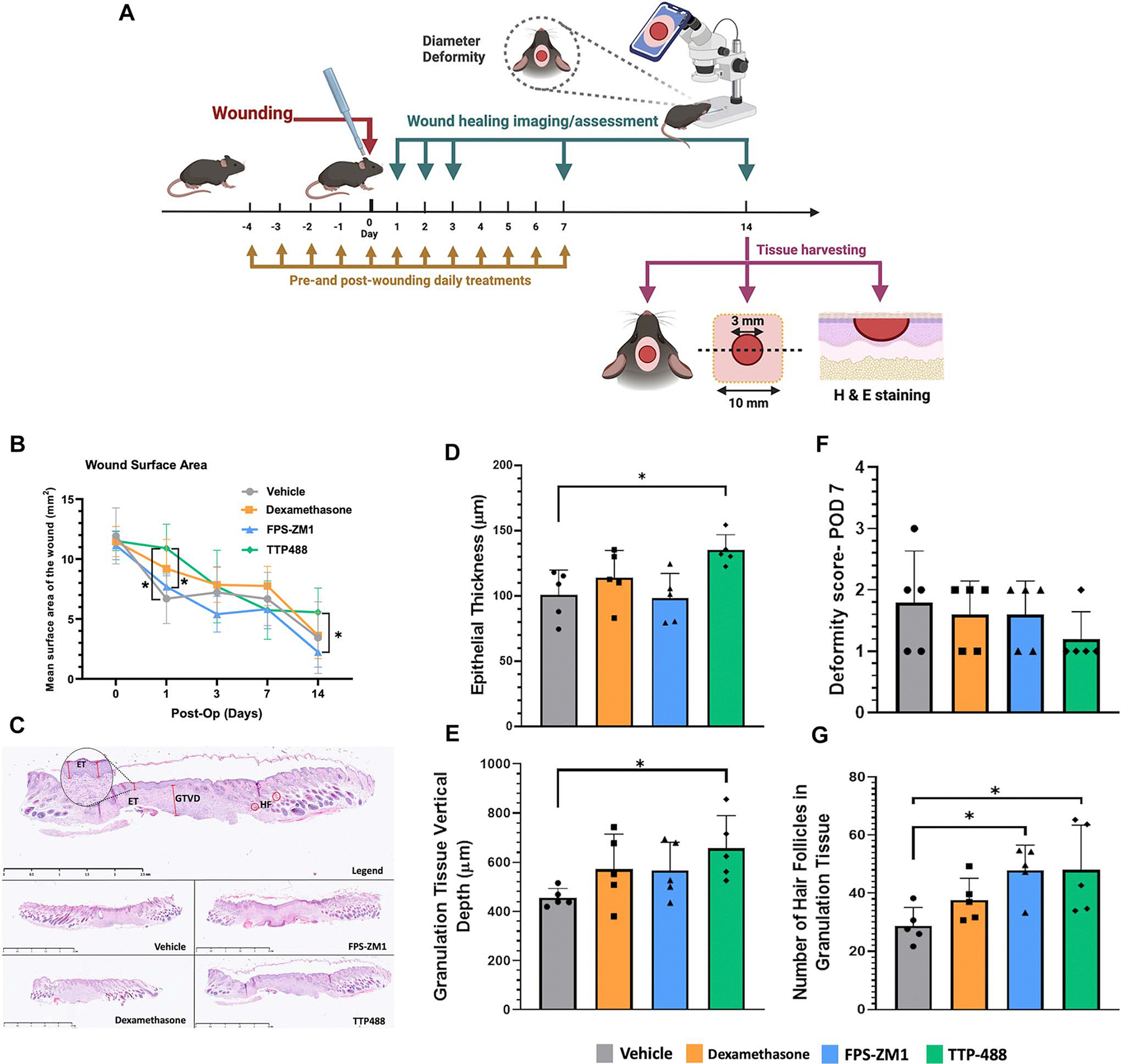
Receptor for Advanced Glycation End products inhibitors do not interfere with wound healing. **A**, Design of the wound healing experiment (open round wounds) illustrating the therapeutic and diagnostic intervention timelines. Mice (n = 5/group) received daily vehicle (100 μL, IP), dexamethasone (100 μg, IP), FPS-ZM1 (2 mg, IP), or TTP488 (100 μg, IP) 4 days before the procedure. **B**, Wound surface area decreased through POD 14 in all groups; however, FPS-ZM1 had the lowest surface areas, indicating better healing compared with the other treatment groups (2-way analysis of variance, F = 128.5, P < .0001; post hoc Dunnett’s multiple comparison test: FPS-ZM1 vs TTP488 on PODs 1 and 14, P = .03, and vehicle vs TTP488, P = .02). **C**, Representative histology of the scalp wounds harvested on POD 14 comparing ET, GTVD, and HF among the treatment groups. **D** and **E**, Quantification of the ET (left graph) and GTVD (right graph) showed significant improvements in the TTP488 group compared with the vehicle. **F**, Assessment of the wound deformity on POD 7 (before hair regrowth) demonstrated that the TTP488 group had the lowest deformity scores, though not statistically significant when compared with the vehicle. **G**, Quantification of the HF inside the granulation tissue region showed a higher number of hair follicles in FPS-ZM1 and TTP488 groups compared with the vehicle. n = 5 mice/group, mean ± SD (*P < .05; **D**-**G**, one-way analysis of variance). ET, epithelial thickness; GTVD, granulation tissue vertical depth; H&E staining, hematoxylin and eosin staining; HF, hair follicles; IP, intraperitoneal; POD, postoperative day; Post-Op, postoperative. Created in BioRender. Dayyani, M. (2026) https://BioRender.com/d8cm24o.

We next evaluated the effect of RAGE inhibitors on the healing of open wounds. All groups showed a linear pattern of decrement in surface area of the wounds through POD 14, with FPS-ZM1 showing the lowest surface areas, indicating better healing compared with the other treatment groups (Figure 3A and 3B) (*P* = .04, FPS-ZM1 vs TTP488). Microscopic evaluation of the wounds (Figure 3C) revealed that the TTP488-treated mice had the highest epithelial thickness (*P* = .02) and granulation tissue vertical depth (*P* = .02) when compared with the vehicle group (Figure 3D and 3E), consistent with better wound healing. Assessment of the wound deformity on POD 7 demonstrated that TTP488-treated mice also had the lowest deformity scores, though not statistically significant when compared with the vehicle (Figure 3F). Interestingly, hair growth evaluation revealed that both FPS-ZM1 and TTP488 modestly improved hair regrowth within the granulation tissue compared with the vehicle (*P* = .02) (Figure 3G).

### Effect of RAGE Inhibitors on Immunotherapy

**FIGURE 4.**
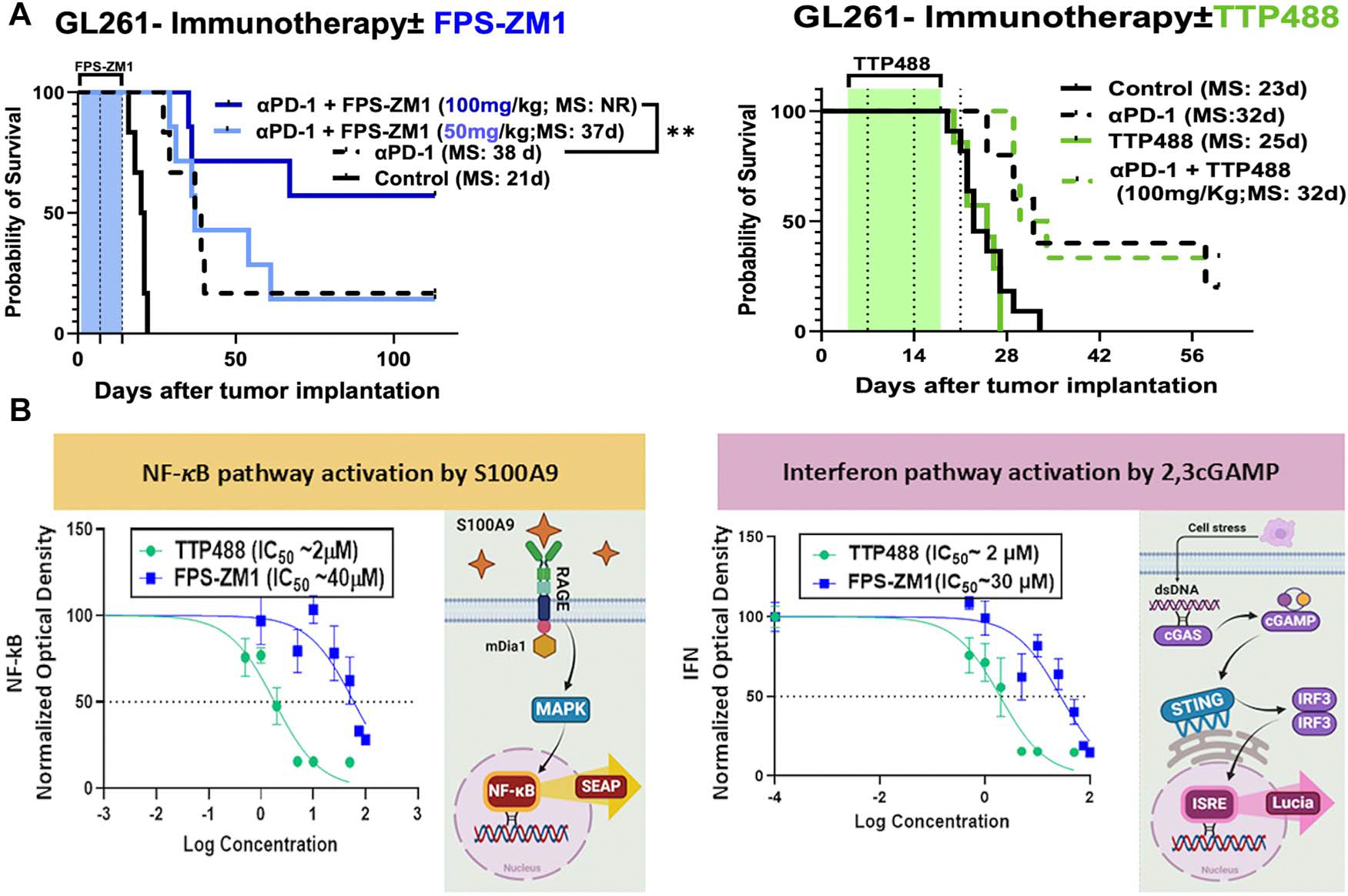
Effect of RAGE inhibitors on immunotherapy and monocyte activation. **A**, Mice bearing orthotopic GL261 gliomas were treated with isotype IgG or anti–PD-1 Ab (vertical dashed lines) and RAGE inhibitors (shaded bars, IP). High doses of FPS-ZM1 (left panel), but not TTP488 (right panel), significantly improved animal survival. Survival was plotted using the Kaplan–Meier method and compared with the log-rank test and Cox regression. **P < .01; n = 7 mice/group. **B**, THP1-Dual assay including Quanti-Blue assay (NF-κB activation by S100A9; left panel) and Renilla luciferase assay (IFN Activation by 2,3cGAMP, right panel) demonstrating more potent inhibition of both pathways by TTP488. αPD-1, anti-programmed death-1; 2,3cGAMP, 2’,3’-cyclic guanosine monophosphate-adenosine monophosphate; cGAS, cyclic GMP-AMP synthasem; dsDNA, double-stranded DNA; GL261, Glioma 261; IC50, half maximal inhibitory concentration; IP, intraperitoneal; IRF3, interferon regulatory factor 3; ISRE, IFN-stimulated response elements; mDia1, mammalian diaphanous related formin 1; MS, median survival; NR, not reached; RAGE, Receptor for Advanced Glycation End products; S100A9, S100 calcium-binding protein A9; SEAP, secreted embryonic alkaline phosphatase; STING, stimulator of interferon genes.

**FIGURE 5.**
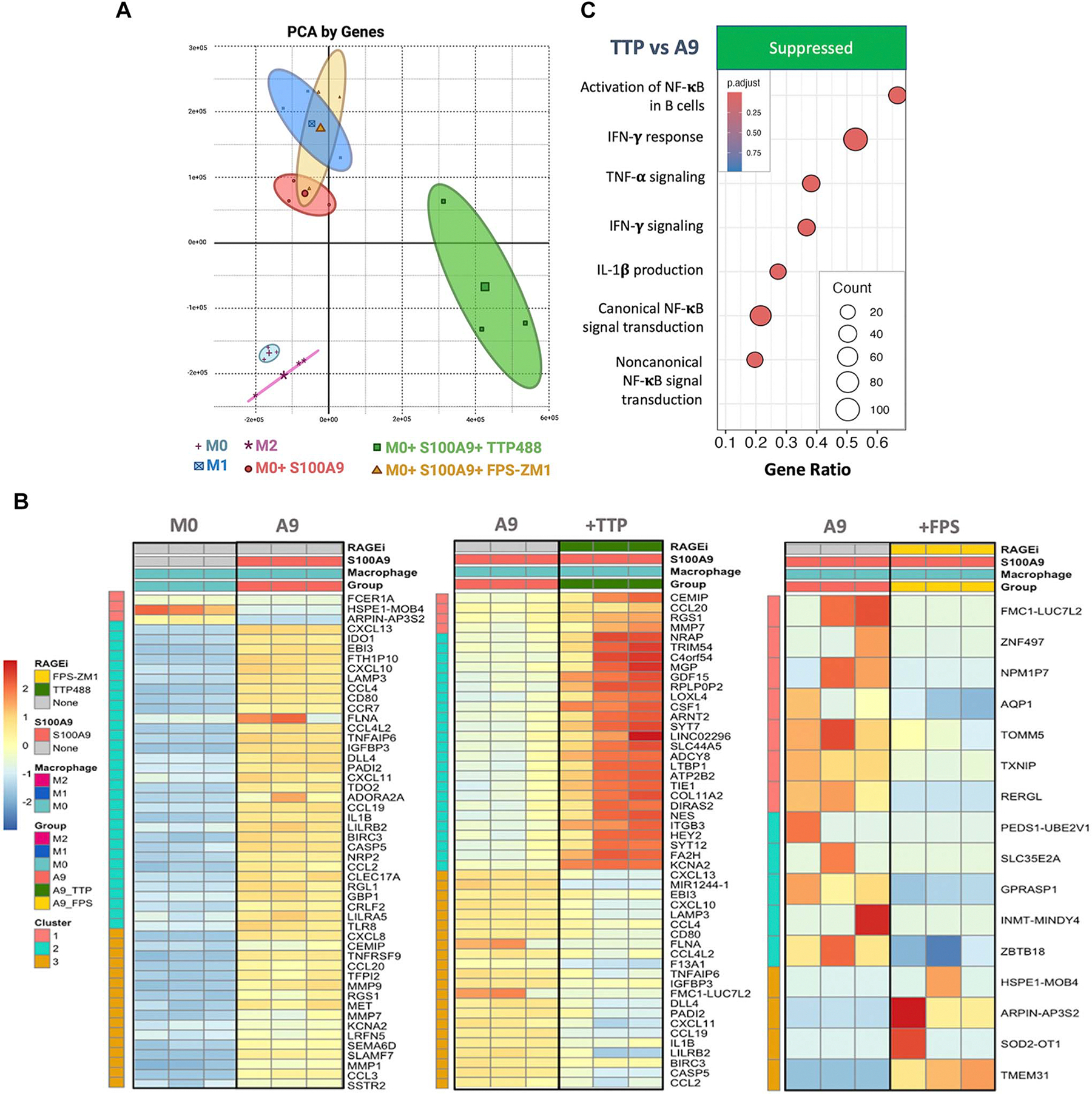

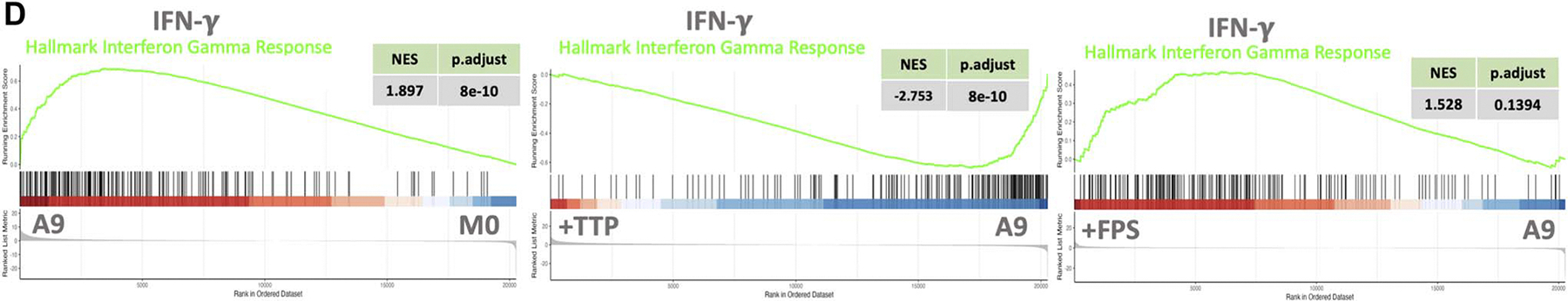
Effects of RAGE inhibitors on macrophage polarization through bulk RNAseq analysis. THP-1 monocytes were differentiated in M0 macrophages and then treated with LPS/IFNγ (M1), IL-4 (M2), S100A9 (500 ng/mL), and each RAGE inhibitor (TTP488: 5 μM, FPS-ZM1 50 μM). **A**, PCA plot demonstrating distinct transcriptomic patterns among various types of macrophages and treatments with RAGE inhibitors. S100A9 stimulation (red) shifts M0 toward an M1-like proinflammatory state, whereas FPS-ZM1 and TTP488 modulate this response, forming distinct clusters. FPS-ZM1 has more similarities with the M1 cluster, whereas TTP488 shifted the cluster further from M1/M0 + S100A9. **B**, Heatmaps demonstrate the comparative gene expression acquired from bulk RNAseq as follows: S100A9 vs M0 (left panel), TTP-488 vs S100A9 (middle panel), and FPS-ZM1 vs S100A9 (right panel). **C**, Pathway analysis revealed inhibition of S100A9-induced NF-kB and IFN-γ proinflammatory signaling pathways by TTP488. **D**, GSEA of IFN-γ for S100A9 vs M0 revealed a significant positive enrichment score of 1.897 (P = 8 × 10^−10^), confirming activation of this pathway by S100A9 (left panel). GSEA of IFN-γ for TTP-488 vs S100A9 showed a significant negative enrichment score of −2.753 (P = 8 × 10^−10^), suggesting suppression of the S100A9-induced IFN-γ pathway by TTP-488 (middle panel). GSEA of the IFN-γ pathway for ranked DE genes for FPS-ZM1 vs S100A9 showed a positive enrichment score in the FPS-ZM1–treated group (NES = 1.52); however, it was not statistically significant (P = .13) (right panel). A9 (S100A9), S100 calcium-binding protein A9; FPS, FPS-ZM1; GSEA, gene set enrichment analysis; LPS, lipopolysaccharide; NES, normalized enrichment score; PCA, principal component analysis; RAGE, Receptor for Advanced Glycation End products; RAGEi, RAGE inhibitor; TTP, TTP488.

To demonstrate the clinical relevance of our findings to brain tumor therapy, we examined the effect of the RAGE inhibitors on the efficacy of immunotherapy in syngeneic Glioma 261 (GL261) and CT-2A orthotopic glioma models. Previously, our group and others showed that dexamethasone abrogates the efficacy of anti–PD-1 therapy in the GL261 orthotopic glioma model^21,36^ (Supplemental Digital Content 1, Supplemental Figure 4A, http://links.lww.com/NEU/F287). Unlike dexamethasone, neither RAGE inhibitor affected the efficacy of anti–PD-1 immunotherapy in 2 glioma models (Supplemental Digital Content 1, Supplemental Figure 4B and 4C, http://links.lww.com/NEU/F287). Because of a positive trend in the overall survival of FPS-ZM1–treated animals (Supplemental Digital Content 1, Supplementary Figure 4B, http://links.lww.com/NEU/F287), we repeated these experiments using higher doses of each RAGE inhibitor. Interestingly, high doses of FPS-ZM1 (ie, 100 mg/kg/day), but not TTP488, enhanced the activity of anti–PD-1 therapy in the GL261 glioma model (Figure 4A). To investigate potential differences in the activity of the RAGE inhibitors, we compared their anti-inflammatory properties in vitro. Compared with FPS-ZM1, TTP488 was a more potent inhibitor of not only S100A9-mediated NF-κB activation but also a more potent inhibitor of the stimulator of interferon genes pathway (Figure 4B). Differences in the anti-inflammatory functions of each RAGE inhibitor were confirmed using bulk RNAseq analysis on polarized human THP-1 monocytes. Our principal component analysis revealed distinct patterns of gene expression for each RAGE inhibitor (Figure 5A and 5B). Gene set enrichment analysis (GSEA) and pathway analysis also showed that TTP488 remarkably dampened S100A9-induced NF-kB (GSEA: normalized enrichment score [NES] = −1.46, *P* = .002) and IFN-γ signaling (GSEA: NES = −2.75, *P* = 8 × 10^−10^), whereas these pathways were not suppressed by FPS-ZM1 (GSEA: NES = 1.13, *P* = .42 for NF-kB; and NES = 1.52, *P* = .13 for IFN-γ) (Figure 5C and 5D, and Supplemental Digital Content 1, Supplemental Figure 5, http://links.lww.com/NEU/F287). Collectively, these findings suggest that, while both RAGE inhibitors were superior to dexamethasone in controlling CE caused by S100A9-RAGE activation, FPS-ZM1 may be better suited for clinical testing with immunotherapies because of lack of inhibitory effects on the interferon pathway, which is beneficial in the context of antitumor immune responses.

## DISCUSSION

In this study, we demonstrate that FPS-ZM1 and TTP488, 2 well-characterized RAGE inhibitors, are as effective as dexamethasone in reducing vasogenic edema after glioma resection in mice. Notably, unlike dexamethasone, RAGE inhibitors did not compromise the efficacy of immunotherapy or impair wound healing. These findings support the potential use of RAGE inhibitors as steroid alternatives for patients with malignant brain tumors undergoing immunotherapy during the perioperative period.

CE and hemorrhage are major complications after neurosurgical procedures.^5^ Although various agents (such as aminoguanidine,^37^ erythropoietin,^38^ nicotinamide adenine dinucleotide phosphate oxidase inhibitors,^39^ Src kinase inhibitors,^40^ simvastatin,^41^ melatonin,^42^ and rosiglitazone^43^) have been evaluated for mitigating CE after SBI, most have shown limited efficacy. To our knowledge, this is the first report demonstrating the anti-inflammatory effects of RAGE inhibitors in a glioma resection model using clinically relevant imaging tools.

Effective therapeutic targeting of CE likely requires modulation of multiple inflammatory pathways. Brain ischemia and injury trigger the release of cytokines and chemokines, which activate pattern recognition receptors,^14^ including RAGE, a multiligand pattern recognition receptor, linked to diabetes, neurodegeneration, inflammation, and cancer.^44–46^ RAGE ligands (S100 calcium-binding protein B, high mobility group box 1, S100A9) exacerbate brain edema and impair sensorimotor function in models of stroke, traumatic brain injury, and tumors.^18,19,21^ We previously showed that RAGE activation by S100A9 contributes to neuroinflammation after SBI.^21^ Using both animal models and resection cavity fluid from craniotomy patients, we confirmed that S100A9 released by infiltrating leukocytes mediates RAGE activation.^21^ Inhibiting S100A9 with tasquinimod or RAGE with FPS-ZM1 was as effective as dexamethasone in reducing CE, supporting the use of S100A9-RAGE inhibitors as steroid alternatives in neurosurgery.^21^

Currently, corticosteroids, primarily dexamethasone, are used to manage CE perioperatively, but they carry significant side effects and interfere with immunotherapy. In the GL261 glioma model, which is responsive to immune checkpoint blockade, we and others have shown that dexamethasone abolishes the efficacy of anti–PD-1 therapy.^21,36^ Similarly, in the immunotherapy-resistant CT-2A model, dexamethasone negates the effects of anti–PD-1 with or without radiotherapy, reduces T-cell numbers via apoptosis, and impairs myeloid, natural killer cell populations, and lymphocyte function.^36^ While tasquinimod also interferes with checkpoint blockade, RAGE inhibitors such as FPS-ZM1 and TTP488 do not.^21^ Here, we demonstrate that both agents suppress the NF-κB pathway by blocking the RAGE-S100A9 axis. However, FPS-ZM1 did not inhibit the IFN pathway activation, which is critical for enhancing antitumor immunity by promoting tumor-infiltrating lymphocyte activity and transforming the tumor microenvironment from immunosuppressive to proinflammatory.^47^ This suggests that RAGE inhibitors, particularly FPS-ZM1, may be ideal steroid alternatives for patients receiving immunotherapy.

In addition to differential impact on the IFN pathway, our bulk RNA-seq analysis of THP-1–polarized macrophages revealed distinct differences in the activity of each RAGE inhibitor. FPS-ZM1 downregulated genes such as AQP1,^48^ GPRASP1,^49,50^ and ZBTB18,^51^ potentially contributing to its anti-inflammatory and antiedematous effects. TTP488, on the other hand, downregulated a broader set of chemokines (CXCL10,^52^ CXCL11,^52^ CXCL13,^53^ CCL2,^54^ CCL4^55^), cytokines (IL-1B^56^), and immune activation genes (lysosome-associated membrane protein 3^57^), thereby reducing central nervous system inflammation, stabilizing the BBB, limiting immune cell infiltration, and ultimately mitigating CE in our glioma model.

### Limitations

Several characteristics of our animal model limit the extrapolation of our findings to human conditions. First, the time course of CE improvement in mouse SBI models was much shorter than that in humans (days vs weeks). Second, we used only one tumor model (CT-2A), which was selected for its MRI visibility and suitability for fluorescence-guided surgery, but may not fully represent the heterogeneity of glioblastoma. Third, the complexity of the glioma resection technique in mice (because of lack of surgical tools, navigation systems, hemostatic agents, etc.) introduced unavoidable variability among the treatment groups. Finally, the inability to perform diffusion-weighted imaging in mice might have slightly limited the precision of CE quantification by MRI.

## CONCLUSION

TTP488 and FPS-ZM1 were as effective as dexamethasone in alleviating CE after tumor resection. Notably, unlike dexamethasone, RAGE inhibitors did not impair the efficacy of immunotherapy or postoperative wound healing. These findings provide a strong rationale for evaluating RAGE inhibitors as potential alternatives to corticosteroids in the postoperative management of patients undergoing tumor resection or other neurosurgical procedures.

## Supplementary Material

Supp **Supplemental Digital Content 1.** Reagents and cell lines.
**Supplemental Digital Content 1.** NF-κB and IFN activation assays.
**Supplemental Digital Content 1.** Surgical brain injury model.
**Supplemental Digital Content 1.** Fluorescence-guided Glioma Resection Survival Surgery (FGRSS).
**Supplemental Digital Content 1:** Supplemental Table 1. MR imaging parameters of the 7-T system.
**Supplemental Digital Content 1:** Supplemental Figure 1. A representative sample from the TTP488 group, segmentation for pre- and postresection volume generation.
**Supplemental Digital Content 1.** Radiomic cluster analysis.
**Supplemental Digital Content 1.** Radiomic feature extraction settings.
**Supplemental Digital Content 1.** Comprehensive wound healing experimental design.
**Supplemental Digital Content 1.** Wound healing assessment protocol.
**Supplemental Digital Content 1:** Supplemental Table 2. Primers used in qPCR.
**Supplemental Digital Content 1.** Bulk RNA-seq data analysis.
**Supplemental Digital Content 1:** Supplemental Figure 2. Neurological examination, component No. 8 of the SNAP score, Baton test.
**Supplemental Digital Content 1:** Supplemental Figure 3. Preliminary findings on wound healing.
**Supplemental Digital Content 1:** Supplemental Figure 4. Effect of dexamethasone and RAGE inhibitors on the efficacy of anti–PD-1 therapy in syngeneic orthotopic glioma models.-
**Supplemental Digital Content 1:** Supplemental Figure 5. GSEA of NF-κB for RAGE inhibitors vs S100A9.

**Supplemental digital content** is available for this article at neurosurgery-online.com.

## ABBREVIATIONS:

BBB: blood-brain barrier
CE: cerebral edema
GL261: Glioma 261
GSEA: gene set enrichment analysis
IP: intraperitoneal
NES: normalized enrichment score
POD: postoperative day
RAGE: Receptor for Advanced Glycation End products
S100A9: S100 calcium-binding protein A9
SBI: surgical brain injury
SNAP: Simple Neuroassessment of Asymmetric Impairment
