# Supplementary material for: RAGE Inhibition Reduces Surgery-Induced Cerebral Edema After Glioma Resection
Source: Neurosurgery. Author manuscript; Available in PMC 2026 Jul 27. (PMC13404757; doi:10.1227/neu.0000000000003987)
Supplement: Supp — lemental Digital Content 1. Reagents and cell lines. Supplemental Digital Content 1. NF-κB and IFN activation assays. Supplemental Digital Content 1. Surgical brain injury model. Supplemental Digital Content 1. Fluorescence-guided Glioma Resection Survival Surgery (FGRSS). Supplemental Digital Content 1: Supplemental Table 1. MR imaging parameters of the 7-T system. Supplemental Digital Content 1: Supplemental Figure 1. A representative sample from the TTP488 group, segmentation for pre- and postresection volume generation. Supplemental Digital Content 1. Radiomic cluster analysis. Supplemental Digital Content 1. Radiomic feature extraction settings. Supplemental Digital Content 1. Comprehensive wound healing experimental design. Supplemental Digital Content 1. Wound healing assessment protocol. Supplemental Digital Content 1: Supplemental Table 2. Primers used in qPCR. Supplemental Digital Content 1. Bulk RNA-seq data analysis. Supplemental Digital Content 1: Supplemental Figure 2. Neurological examination, component No. 8 of the SNAP score, Baton test. Supplemental Digital Content 1: Supplemental Figure 3. Preliminary findings on wound healing. Supplemental Digital Content 1: Supplemental Figure 4. Effect of dexamethasone and RAGE inhibitors on the efficacy of anti–PD-1 therapy in syngeneic orthotopic glioma models.- Supplemental Digital Content 1: Supplemental Figure 5. GSEA of NF-κB for RAGE inhibitors vs S100A9. [file NIHMS2190489-supplement-Supp.docx]

**Supplemental Digital Contents**

**RAGE Inhibition Reduces Surgery-Induced Cerebral Edema Following Glioma Resection**

Mojtaba Dayyani, Aleksandr Filippov, Ian Zhang, Joseph Georges, Zaan Saeed, Jillyn Turunen, Nick Sobhanian, Huiling Yuan, Massimo D’Apuzzo, Yue Hao, Michael E. Berens, Leying Zhang, Jana Portnow, Behnam Badie^*^

Table of Contents

[Supplemental digital content 1: Reagents and cell lines 2](#_Toc207713058)

[Supplemental digital content 2: NF-κB and IFN activation assays 3](#_Toc207713059)

[Supplemental digital content 3: Surgical Brain injury model 4](#_Toc207713060)

[Supplemental digital content 4: Fluorescence-guided Glioma Resection Survival Surgery (FGRSS) 5](#_Toc207713061)

[Supplemental digital content 5, supplemental Table 1. MR imaging parameters of the 7-T system 6](#_Toc207713062)

[Supplemental digital content 6, supplemental Figure 1. A representative sample from the TTP488 group segmentation for pre- and post-resection volume generation. 7](#_Toc207713063)

[Supplemental digital content 7. Radiomic Cluster Analysis 8](#_Toc207713064)

[Supplemental digital content 8: Radiomic Feature Extraction Settings^1^ 9](#_Toc207713065)

[Supplemental digital content 9: Comprehensive wound healing experimental design 10](#_Toc207713066)

[Supplemental digital content 10: Wound healing assessment protocol 11](#_Toc207713067)

[Supplemental digital content 11, supplemental Table 2. Primers used in qPCR 12](#_Toc207713068)

[Supplemental digital content 12, Bulk RNA-seq data analysis 13](#_Toc207713069)

[Supplemental digital content 13, supplemental Figure 2. Neurological examination, component No.8 of the SNAP score, Baton test. 14](#_Toc207713070)

[Supplemental digital content 14, supplemental Figure 3: Preliminary findings on wound healing 15](#_Toc207713071)

[Supplemental digital content 15, supplemental Figure 4. Effect of dexamethasone and RAGE inhibitors on the efficacy of anti-PD-1 therapy in syngeneic orthotopic glioma models. 16](#_Toc207713075)

[Supplemental digital content 16, supplemental Figure 5. GSEA of NF-𝛋B for RAGE inhibitors vs S100A9 17](#_Toc207713076)

# **Supplemental digital content 1: Reagents and cell lines**

THP1-Dual cells were purchased from InvitroGen and were cultured in RPMI 1640 medium supplemented with 10% FBS, 10 mM HEPES buffer solution, and 100 nM penicillin/streptomycin, maintaining them at 37°C and 5% CO2. Sterility, stability, and authenticity of the cell lines were confirmed biannually by IDEXX Bioanalytics (ME, USA). TTP488 and FPS-ZM1 were purchased from MedChemExpress (NJ, USA). For in vivo experiments, TTP488 and FPS-ZM1 were dissolved in vehicle containing 10% DMSO, 40% PEG-300, 5% Tween-80, and 45% PBS. S100A9 was purchased from ProSpec-Tany TechnoGene Ltd. (NJ, USA).

# **Supplemental digital content 2: NF-κB and IFN activation assays**

**THP-1 Quanti-Blue assay (NF-κB activation)**

THP-Dual cells were seeded in a 96-well plate at a density of 2,000 cells per well and incubated at 37°C with 5% CO₂ for 24 hours. Cells were then incubated with different doses of TTP488 (0.5–50 μM) and FPS-ZM1 (0.5–50 μM) for 1 hour before adding S100A9 (500 ng/mL). After 24 hours of incubation, 20 μl of the samples were added to 180 μl of QUANTI-Blue™ Solution and 30 minutes later, the optical density (OD) was measured at 625 nm using a microplate reader (SpectraMax® iD3).

**THP-1 Renilla Luciferase assay (IFN activation)**

Cell treatments were similar to the Quanti-Blue Assay except that 2,3-cGAMP (20μg/ml) was used instead of S100A9 to activate the cells. After 24 hours of incubation, 20µl of each sample was added to an opaque plate containing 100µl of Renilla Luciferase reagent, before measuring luminescence emission.

#

# **Supplemental digital content 3: Surgical Brain injury model**

Mice (C57BL/6 J, n=3/ group) weighing 20–25 g, received TTP488 (100 µg, IP), dexamethasone (100 µg, IP), or vehicle (100 µl, IP) perioperatively (day -4 pre-op to day 7 post-op). Mice were then anesthetized by ketamine (132 mg/kg, IP) and xylazine (8.8 mg/ kg, IP). A 2-mm craniectomy was performed with a drill, and a 1-mm3 portion of frontal brain tissue was excised using a disposable biopsy punch instrument (Integra Life Sciences Services, Integra York PA, Inc.) and micro-scissors. CE was quantified with serial brain MRIs on postoperative days 1, 2, 3, and 7

# **Supplemental digital content 4: Fluorescence-guided Glioma Resection Survival Surgery (FGRSS)**

Mice (C57BL/6J) harboring 10-day-old CT-2A gliomas were imaged with MRI to measure tumor size. Mice with superficial tumors that were amenable to resection were stratified into four uniform treatment groups: vehicle, dexamethasone, TTP488, or FPS-ZM1 (n=4-6 mice/group). Treatments began 4 days prior to fluorescence-guided glioma resection survival surgery (FGRSS) with a novel U-arm 3D-printed neurosurgical platform (UNP) and continued for 7 days after the procedure.

The animals anesthetized with an intraperitoneal (IP) injection of Ketamine and Xylazine and/or isoflurane inhalation. Once the depth of anesthesia is ensured, the animal transferred to the clean surgery area and the animal's head will be secured to the stereotaxic apparatus. Eye lubricant applied to the animal's eyes to prevent dryness. After hair removal and skin sterilization with povidone-iodine and isopropyl alcohol, a small midline incision (≈8-10 mm) was made from the inter-ocular line to the bregma with a disposable sterile blade.

The old burr hole that was made for the tumor implantation at stereotactic coordinates (1 mm anterior and 2 mm right lateral of the bregma), was identified and gradually enlarged with cautious drilling more inclined to the lateral side to avoid bleeding from the superior sagittal sinus. After preparing a burr hole with an average diameter of 4-5 mm, we used 1.5 and 2 mm micro currets (Fine Science Tools, Heidelberg, Germany) to excise the tumor tissue with the guidance of the pre-op MRIs available at the time of surgery. Also, we used fluorescence-guided surgery (FGS) to enhance the surgical precision by enabling real-time visualization of the tumor tissue. Fluorescein dye (5 mg/kg) was administered intraperitoneally 30 minutes prior to surgery. A digital fluorescence microscope (DinoLite, 20X magnification) was used to detect fluorescein-labeled tumor under blue-light conditions.

During the entire surgery, bleeding was controlled with peroxide, bone vax (©ETHICON, INC. 2013) and Surgicel (©ETHICON, INC. 2013) as appropriate based on the source of the bleeding. After resection, the tumor cavity was irrigated with warm saline solution, and filled with Surgicel. Once we ensured there is no bleeding, the wound was closed using skin glue and buprenorphine (0.05 mg/kg, SC) was administered for analgesia. In the recovery phase, each mouse was placed on the warm blanket in a reverse Trendelenburg position to expedite recovery.

# **Supplemental digital content 5, supplemental Table 1. MR imaging parameters of the 7-T system**

| Pulse Sequence | T1-weighted imaging (± GBCA) | T2-weighted imaging | T2-FLAIR |
| --- | --- | --- | --- |
| Repetition Time (TR) (ms) | 1312 ± 0 | 3000 ± 0 | 4500 ± 500 |
| Echo Time (TE) (ms) | 11 | 45 | 45 |
| Inversion Time (TI) (ms) | N/A | N/A | 1000 |
| Pixel size (µm) | 70.5228 ± 0.0613 | 75.7737 ± 5.19 | 75.7737 ± 5.19 |
| Section thickness (mm) | 0.7 | 0.7 | 0.7 |
| Slice Gap (mm) | 0.1 | 0.1 | 0.1 |
| Views | 256 | 256 | 256 |
| Samples | 256 | 238 | 238 |
| FOV | 18 | 18 | 18 |
| FOV Ratio | 1.0 | 1.0 | 1.0 |
| Slice count | 16 | 16 | 16 |
| Scan time | 1:25 (mm:ss) | 1:45 (mm:ss) | 2:20 – 2:55 (mm:ss) |
| Echo Train | 4 | 7 | 7 |
| Echo Spacing | 11 | 15 | 15 |
| Fat Saturation | Enabled | Enabled | Enabled |

Abbreviation: GBCA = Gadolinium-based Contrast Agents; FLAIR = fluid-attenuated inversion recovery

# **Supplemental digital content 6, supplemental Figure 1. A representative sample from the TTP488 group segmentation for pre- and post-resection volume generation.**


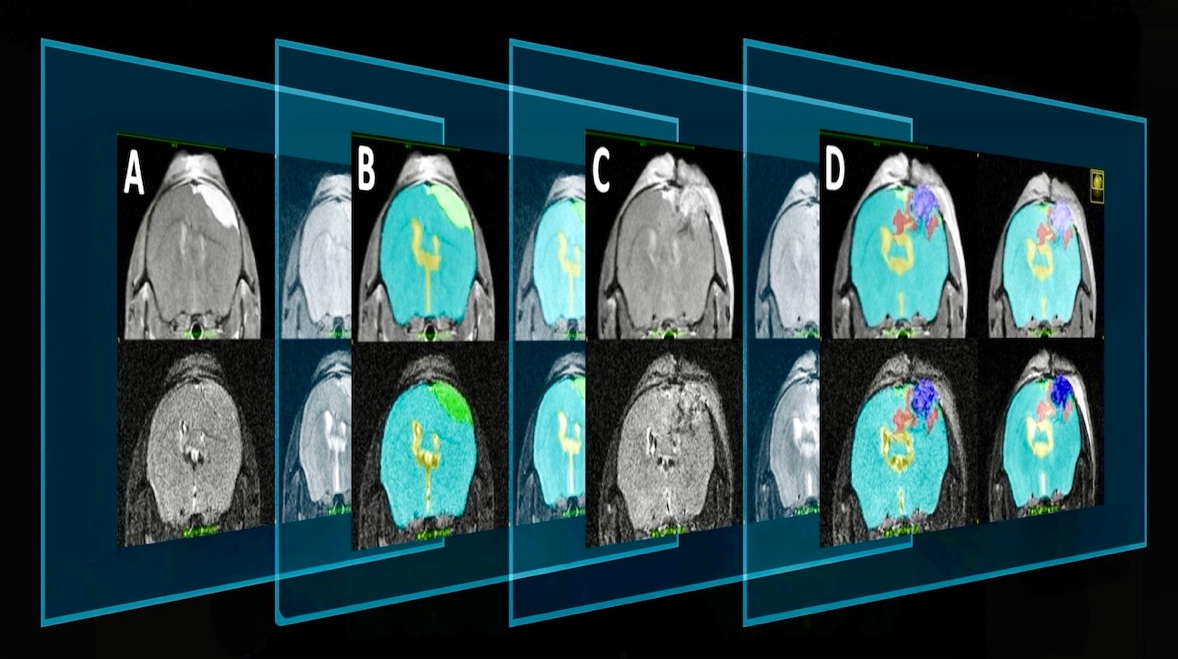


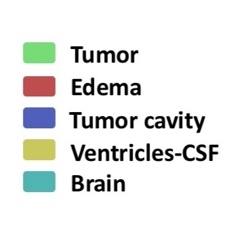


**Figure shows pre-resection axial MRI (A) and its correspondent volume generated image (B), followed by post-resection axial MRI (C) and its correspondent volume generated image (D).** Volumes/masks were manually segmented in the following order with the accompanying heuristics: T1-hyperintense signal: In the pre-resection context, meaningful T1 hyperintense signals were rarely seen. T1-hyperintense signals were marked in the T1-pre contrast images to account for iatrogenic effects such as the resection cavity itself, the T1 hyperintense signal secondary to surgical trauma, Surgicel placement, and cranial disruption.

1. Enhancing Tumor: Remaining T1-hyperintense signals in the T1-post contrast images were considered “enhancing tumor” as the native T1-hyperintense signals were accounted for.
2. Ventricles and CSF: To prevent errant calculation of edema volumes, the ventricles (lateral, third, and fourth) were segmented based on their anatomical location and comparisons between T2-weighted and T2-FLAIR signals.
3. Edema: The remaining T2 hyperintense signals not attributable to anything else were marked as edema.
4. Brain: Remaining tissue in the cranial cavity was marked as brain in order to calculate ratios relative to brain tissue.

# **Supplemental digital content 7. Radiomic Cluster Analysis**

**Radiomic Feature clustering of Edema changes after SBI on POD1.**

3-Dimensional Shape and Feature-based radiomic parameters using the T2-weighted image and label for CE were extracted using PyRadiomics1 with the feature extraction settings mentioned in **Supplementary material 3**. Three mice from each treatment group on POD1 were analyzed. After removing features with zero variance and scaling the entire feature set, unsupervised z-scaled hierarchical clustering revealed a closer relationship between Dexamethasone and TTP488 compared to the vehicle, indicating the similarity of the radiologic phenotypes for these two agents.

# **Supplemental digital content 8: Radiomic Feature Extraction Settings^1^**

imageType:
 Original: {}
 LBP2D: {}
 LoG:
 sigma: [1.0, 2.0, 3.0, 4.0]
 Wavelet: {}
featureClass:
 shape:
 firstorder:
 ['InterquartileRange', 'Kurtosis', '10Percentile', 90Percentile', 'MeanAbsoluteDeviation', 'Median', Energy', 'RootMeanSquared','Skewness', 'Variance', 'Entropy']
 glcm:
 ['Imc1', 'Idm', 'InverseVariance', 'SumEntropy', 'Imc2', 'DifferenceVariance', 'ClusterProminence', 'Idn', 'Idmn']
 ngtdm:
 ['Coarseness', 'Contrast', 'Busyness', 'Complexity']
 glrlm:
 ['ShortRunEmphasis', 'ShortRunHighGrayLevelEmphasis','GrayLevelNonUniformity', 'LongRunLowGrayLevelEmphasis', 'HighGrayLevelRunEmphasis', 'RunPercentage']
 gldm:
setting:
 binWidth: 25
 force2D: True
 normalize: True

label: 1
 minimumROISize: 1
 interpolator: 'sitkBSpline'
 resampledPixelSpacing:
 voxelArrayShift: 200 # only shift if necessary
 geometryTolerance: 0.05
 distances: [1,2,3]

#

# **Supplemental digital content 9: Comprehensive wound healing experimental design**

In a pilot wound healing study, mice were randomized into three groups [vehicle (100 μl IP/ day), TTP488 (100 μg IP/ day), or dexamethasone (100 μg IP/day, n=4/group)]. Scalps were shaved, prepped, and a small linear midline incision (≈8-10 mm) was made, which was then closed using anskin glue (GLUture®, Zoetis Inc.). We obtained color images of the wounds immediately after skin incision (day 0) and on POD 1, 3, 7, and 14. The images of POD 7 were selected (the most extended period before hair regrowth) to assess wound redness, dehiscence, and deformity by four observers who were blind to the treatment assignments. To ensure the consistency of evaluation and scoring among our examiners, inter-rater agreement was measured using inter-class correlation coefficient (ICC) method.

To further evaluate the effect of RAGE inhibitors on wound healing, we designed a more sophisticated experiment in which mice were randomized into four groups [vehicle (100 μl IP/ day), dexamethasone (100 μg IP/day, n=5/group), FPS-ZM1 (2 mg IP/ day), and TTP488 (100 μg IP/ day)]. The scalp was shaved, prepped, and a round wound (≈3 mm diameter) was generated with a skin biopsy punch (Integra Life Sciences Services, Integra York PA, Inc.) over the bregma.^2^ To assess secondary wound closure, digital images of the wounds were obtained immediately after skin incision (day 0) and on POD 1, 3, 7, and 14. The images of POD 7 and POD 14 were selected to quantify wound surface area, deformity, and hair growth using Image J software (1.54d, Wayne Rasband and contributors, National Institutes of Health, USA).

On POD 14, all scalp wounds were harvested, fixed in 10% neutral buffered formalin, embedded in paraffin, sectioned, and digitized for microscopic evaluations. A pathologist who was blinded to the treatment groups examined wound healing by measuring the following criteria: epithelial thickness (ET) represented the area above the granulation tissue; granulation tissue vertical depth (GTVD) constituted the maximal vertical extent of granulation tissue seen within the analyzed level; and hair follicles (HF) in the granulation tissue.

# **Supplemental digital content 10: Wound healing assessment protocol**

Immediately after skin incision (day 0) and on post-operative days 1, 3, 7, and 14, we mounted an iPhone 13 Pro onto a dissecting microscope (Wild M5A, Switzerland) with a Celestron optic adaptor (Torrance, CA, US) and captured sequential color images under 12X and 25X magnification with fixed lighting and color settings. Before the re-growth of the scalp hair, four researchers who were blinded to the group assignments of the experiment, used a visual assessment protocol to rank the wound healing based on the relative comparison of the three quantifiable criteria, including redness, dehiscence, and deformity.

For all three items, we defined a numeric rating scale including: Redness (score 0 indicates no redness of the wound bed, scores 1, 2, and 3 indicate a mild, moderate and severe redness of the wound bed, respectively. One extra score is added if wound edges are markedly inflamed as well); dehiscence (score 0 indicate fully closed wound, scores 1, 2, 3 indicate 25%, 50%, and 75% of the wound is remained open, respectively); deformity (score 0 indicates a completely straight wound line, score 1 indicates a mild malformed wound, score 2 indicates a mild malformed wound along with skin induration, and score 3 indicates a moderate or severe S shaped deformity with a skin contracture or skin induration).

Because dehiscence is clinically more important than other parameters, we weighted it twice as high as other scores for all mice in all groups. Wounds were considered closed if moist granulation tissue was no longer apparent, and the wound appeared covered with new epithelium.

# **Supplemental digital content 11, supplemental Table 2. Primers used in qPCR**

| GAPDH-f | ACGGCAAATTCAACGGCACAGTCA |
| --- | --- |
| GAPDH-r | CATTGGGGGTAGGAACACGGAAGG |
| IL1β-f | AGGGCTGTCTGGAGTCCTC |
| IL1β-r | GACCAGCCGCCGCCGCAGG |
| m-CCL4 MIP1β-F | CAACACCATGAAGCTCTGCG |
| m-CCL4 MIP1β-R | GCCACGAGCAAGAGGAGAGA |
| m-CCL3/MIP-1α-F | TGAAACCAGCAGCCTTTGCTC |
| m-CCL3/MIP-1α-R | AGGCATTCAGTTCCAGGTCAGTG |

# **Supplemental digital content 12, Bulk RNA-seq data analysis**

THP-1 monocytes were first differentiated into M0 macrophages (PMA, 25 ng/mL for 48 hours). The PMA media was replaced with fresh media for 24 hours before treating the cells with LPS (50 ng/mL) + IFN-γ (5 ng/mL, M1), IL-4 (25 ng/mL, M2), S100A9 (500 ng/mL), and each RAGE inhibitor (TTP488: 5μM; FPS-ZM1: 50 μM) for another 24 hours. Transcriptomics FASTQ data quality was assessed using FastQC v0.11.8,^3^ and preprocessed using fastp.^4^ Reads were aligned to the human reference genome (hg38) using HISAT2.^5^ StringTie^6^ was adopted for fast and highly efficient assembly of RNA-Seq alignments into potential transcripts. Ballgown^7^ was applied to facilitate flexible differential expression analysis of RNA-seq data. Gene-level expression quantification was normalized using DESeq2 v1.46.0.^8^ Principal component analysis (PCA) revealed the overall transcriptomic similarity and differences between the six groups. Pairwise differential expression analyses were performed for M1 vs M0, M2 vs M0, A9 vs M0, FPS vs A9, and TTP vs A9. Heatmap figures of differentially expressed genes were plotted using pheatmap v1.0.12.^9^ Statistically overrepresented gene ontology terms and pathways were identified using clusterProfiler v4.14.6.^10^Gene Set Enrichment Analyses (GSEA) were performed using clusterProfiler v4.14.6 with genome-wide annotation for human org.Hs.eg.db and the Molecular Signatures Database^11^ R implementation msigdbr v10.0.1.^12^

# **Supplemental digital content 13, supplemental Figure 2. Neurological examination, component No.8 of the SNAP score, Baton test.**


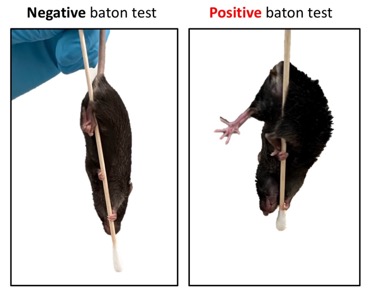


# **Supplemental digital content 14, supplemental Figure 3: Preliminary findings on wound healing**


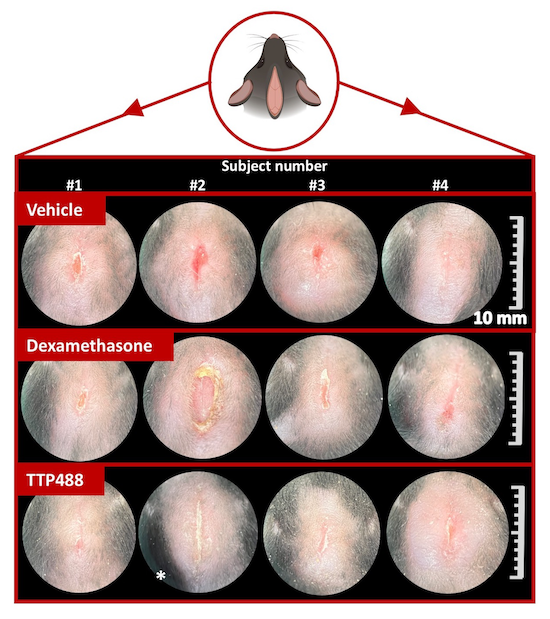

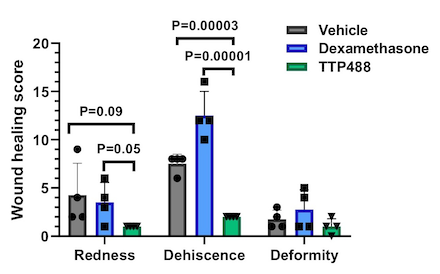


**A**

**B**

**C**


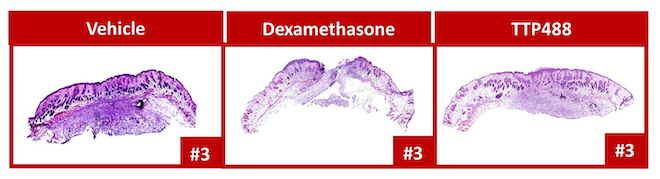


**Quantification of the wound healing:** **A,** Images of the scalp wounds under the surgical microscope on POD 7 demonstrate rapid wound healing in TTP488 group. **B,** TTP488 group exhibited lower dehiscence and redness compared to the control and dexamethasone groups. n=4 mice/treatment group, mean± SD (One-way ANOVA). **C,** Representative histology of the scalp wounds harvested on POD 14 demonstrating delayed epithelialization in the Dexamethasone group but not in the TTP488-treated mice.

To assess the consistency of evaluation and scoring among the examiners, the ICC was calculated for all groups and revealed the following values: vehicle, ICC= 0.96 [95% CI, 0.76-0.99]; dexamethasone, ICC= 0.98 [95% CI, 0.87-1.0]; and TTP488, ICC= 0.92 [95% CI, 0.44-0.99]. The ICC values represent 92%, 96%, and 84% of agreements among the examiners for each group, respectively.

# **Supplemental digital content 15, supplemental Figure 4. Effect of dexamethasone and RAGE inhibitors on the efficacy of anti-PD-1 therapy in syngeneic orthotopic glioma models.**


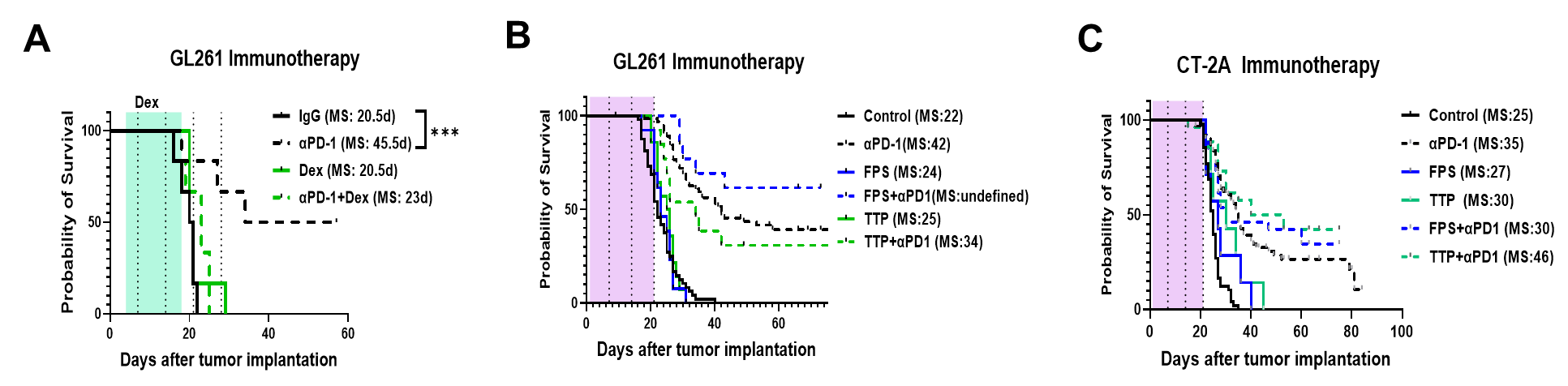


Mice bearing orthotopic GL261 were treated with weekly isotype IgG (100 µg, IP) or anti-PD1 Ab (100 µg/IP) (dashed lines) one week after tumor implantation. **A,** Daily dexamethasone (Dex, 100 μg/IP) administered for 21 days (green shade) dampened the response to anti-PD-1 immunotherapy (n=7/group). Data is modified from the original^13^. **B,** In addition to anti-PD-1, GL261-bearing mice were also treated with either TTP488 (TTP, 100 μg/day/IP) or FPS-ZM1 (FPS, 2 mg/day/IP) for 21 days (purple shade). Neither RAGE inhibitor impacted the efficacy of anti-PD-1 immunotherapy. Data was pooled from three different experiments (n = 20-30/group). **C,** Neither RAGE inhibitor impacted the efficacy of anti-PD-1 immunotherapy on orthotopic CT-2A gliomas. Data was pooled from three different experiments (n = 20-30/group).

# **Supplemental digital content 16, supplemental Figure 5. GSEA of NF-𝛋B for RAGE inhibitors vs S100A9**


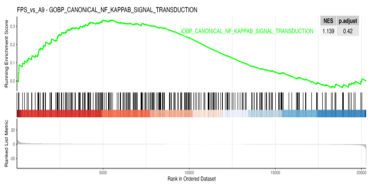


**+FPS**

**A9**

**NF-𝛋B**

**B**


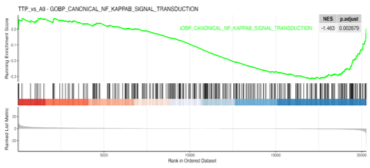


**+TTP**

**A9**

**NF-𝛋B**

**A**

1. GSEA of NF-𝛋B for TTP-488 vs S100A9 showed a significant negative enrichment score of -1.463, indicating the suppression of the NF-𝛋B pathway in the TTP-488-treated group.
2. GSEA of NF-𝛋B for FPS-ZM1 vs S100A9 showed a positive enrichment score of 1.139.

**References**:

1. van Griethuysen JJM, Fedorov A, Parmar C, et al. Computational Radiomics System to Decode the Radiographic Phenotype. *Cancer Research.* 2017;77(21):e104-e107.

2. Reid RR, Said HK, Mogford JE, Mustoe TA. The future of wound healing: pursuing surgical models in transgenic and knockout mice. *J Am Coll Surg.* 2004;199(4):578-585.

3. Andrews S. FastQC: a quality control tool for high throughput sequence data. In:2010.

4. Chen S, Zhou Y, Chen Y, Gu J. fastp: an ultra-fast all-in-one FASTQ preprocessor. *Bioinformatics.* 2018;34(17):i884-i890.

5. Pertea M, Kim D, Pertea GM, Leek JT, Salzberg SL. Transcript-level expression analysis of RNA-seq experiments with HISAT, StringTie and Ballgown. *Nat Protoc.* 2016;11(9):1650-1667.

6. Shumate A, Wong B, Pertea G, Pertea M. Improved transcriptome assembly using a hybrid of long and short reads with StringTie. *PLoS Comput Biol.* 2022;18(6):e1009730.

7. Frazee AC, Pertea G, Jaffe AE, Langmead B, Salzberg SL, Leek JT. Ballgown bridges the gap between transcriptome assembly and expression analysis. *Nat Biotechnol.* 2015;33(3):243-246.

8. Love MI, Huber W, Anders S. Moderated estimation of fold change and dispersion for RNA-seq data with DESeq2. *Genome Biol.* 2014;15(12):550.

9. Kolde R. pheatmap: Pretty Heatmaps. In:2018.

10. Yu G, Wang LG, Han Y, He QY. clusterProfiler: an R package for comparing biological themes among gene clusters. *OMICS.* 2012;16(5):284-287.

11. Liberzon A, Subramanian A, Pinchback R, Thorvaldsdottir H, Tamayo P, Mesirov JP. Molecular signatures database (MSigDB) 3.0. *Bioinformatics.* 2011;27(12):1739-1740.

12. Dolgalev I. msigdbr: MSigDB Gene Sets for Multiple Organisms in a Tidy Data Format. In:2025.

13. Liu S, Song Y, Zhang IY, et al. RAGE Inhibitors as Alternatives to Dexamethasone for Managing Cerebral Edema Following Brain Tumor Surgery. *Neurotherapeutics : the journal of the American Society for Experimental NeuroTherapeutics.* 2022;19(2):635-648.
